# Long noncoding RNA *PVT1* indicates a poor prognosis of gastric cancer and promotes cell proliferation through epigenetically regulating p15 and p16

**DOI:** 10.1186/s12943-015-0355-8

**Published:** 2015-04-12

**Authors:** Rong Kong, Er-bao Zhang, Dan-dan Yin, Liang-hui You, Tong-peng Xu, Wen-ming Chen, Rui Xia, Li Wan, Ming Sun, Zhao-xia Wang, Wei De, Zhi-hong Zhang

**Affiliations:** Clinical Medical Examination Center, Northern Jiangsu People’s Hospital, Yangzhou, Jiangsu PR China; Department of Biochemistry and Molecular Biology, Nanjing Medical University, Nanjing, Jiangsu PR China; Cancer Research and Therapy Center, The Second Affiliated Hospital of Southeast University, Nanjing, 210029 Jiangsu PR China; Nanjing Maternity and Child Health Care Institute, Nanjing Maternity and Child Health Care Hospital Affiliated with Nanjing Medical University, Nanjing, 210029 China; Department of Oncology, First Affiliated Hospital of Nanjing Medical University, Nanjing, Jiangsu PR China; Department of Oncology, Second Affiliated Hospital of Nanjing Medical University, Jiangjiayuan Road, Nanjing, 210011 Jiangsu PR China; Departments of Pathology, First Affiliated Hospital of Nanjing Medical University, Nanjing, Jiangsu PR China

**Keywords:** *PVT1*, Cell proliferation, p15, p16, Gastric cancer

## Abstract

**Background:**

Mounting evidence indicates that long noncoding RNAs (lncRNAs) could play a pivotal role in cancer biology. However, the overall biological role and clinical significance of *PVT1* in gastric carcinogenesis remains largely unknown.

**Methods:**

Expression of *PVT1* was analyzed in 80 GC tissues and cell lines by qRT-PCR. The effect of *PVT1* on proliferation was evaluated by MTT and colony formation assays, and cell apoptosis was evaluated by Flow-cytometric analysis. GC cells transfected with sh*PVT1* were injected into nude mice to study the effect of *PVT1* on tumorigenesis in vivo. RIP was performed to confirm the interaction between *PVT1* and EZH2. ChIP was used to study the promoter region of related genes.

**Results:**

The higher expression of *PVT1* was significantly correlated with deeper invasion depth and advanced TNM stage. Multivariate analyses revealed that *PVT1* expression served as an independent predictor for overall survival (p = 0.031). Further experiments demonstrated that *PVT1* knockdown significantly inhibited the proliferation both in vitro and in vivo. Importantly, we also showed that *PVT1* played a key role in G1 arrest. Moreover, we further confirmed that *PVT1* was associated with enhancer of zeste homolog 2 (EZH2) and that this association was required for the repression of p15 and p16. To our knowledge, this is the first report showed that the role and the mechanism of *PVT1* in the progression of gastric cancer.

**Conclusions:**

Together, these results suggest that lncRNA *PVT1* may serve as a candidate prognostic biomarker and target for new therapies in human gastric cancer.

**Electronic supplementary material:**

The online version of this article (doi:10.1186/s12943-015-0355-8) contains supplementary material, which is available to authorized users.

## Introduction

Gastric cancer (GC) is the second leading cause of cancer death and the most common gastrointestinal malignancy in East Asia [1]. Unfortunately, gastric cancer is often diagnosed at advanced stage in most patients and the prognosis is still very poor [2]. Despite efforts in multiple fields, there has been little improvement in early diagnosis and treatment of gastric cancer. Thus, a better understanding of the mechanisms underlying GC development and progression is essential for improving prevention, diagnosis and treatment of this disease.

Recent advances in whole-genome sequencing technology have led to the discovery of a new type of regulation gene, i.e. long noncoding RNAs (lncRNAs), which are more than 200 bases in length and unable to be translated into proteins. Emerging evidence suggests that lncRNAs may play critical roles in cellular development, differentiation, and many other biological processes [3-5]. The dysregulation of lncRNAs has been shown in various types of cancer including gastric cancer [6-9]. Molecular mechanisms of lncRNAs are diverse. They have been shown to regulate gene expression at multiple levels, including chromatin modification, transcription and post-transcriptional processing. For example, lncRNAs regulate gene transcription through recruiting transcription factors to their target gene promoters, therefore activating gene expression [10]. Moreover, they can also block binding of transcription factors, potentially via formation of RNA-DNA-Triplexes [11]. In mammalian cells, lncRNA *HOTAIR* interacts with PRC2 (Polycomb Repressive Complex 2) to induce heterochromatin formation in specific gene loci leading to inactivation of target genes [12]. Furthermore, lncRNAs can modulate gene expression in post-transcriptionally levels [13-15].

Increasing amount of evidence suggests that numerous lncRNAs have been identified to regulate gene expression through binding to PRC2 in various biological processes, especially in cancer [16,17]. PRC2 is involved in many biological processes, including differentiation, maintaining cell identity and proliferation, and stem-cell plasticity [18]. EZH2, a key catalytic subunit of PRC2 (EZH2, SUZ12 and EED), functions as a histone methyltransferase that specifically induces histone H3 lysine 27 trimethylation (H3K27me3) to target genes [19]. Overexpression of EZH2 is a marker of advanced and metastatic disease in numerous cancers, including bladder cancer [20], gastric cancer [21], lung cancer [22], cervical cancer [23] and hepatocellular carcinoma [24]. So far, long non-coding RNAs are becoming recognized as important participants in PRC2 function.

*PVT1* oncogene (*PVT1*) encodes a long noncoding RNA and maps to chromosome 8q24 [25]. Alvarez ML et al. has showed that *PVT1* may mediate the development and progression of diabetic nephropathy through mechanisms involving ECM accumulation [26]. Amplification of *PVT1* is one of the most frequent events in a variety of malignant diseases, including colorectal cancer [27], serous ovarian and breast cancers [28], and has been associated with reduced survival duration in patients. To sum up, the dysregulation of *PVT1* involves in a wide variety of diseases, especially in tumors. However, the function role and molecular mechanism of *PVT1* in gastric cancer remains unclear.

In the current study, we showed that *PVT1* was markedly increased in gastric cancer tissues compared with adjacent non-tumor tissues and could be served as an independent predictor for overall survival in gastric cancer. In addition, *PVT1* could regulate gastric cancer cell growth both in vitro and in vivo. Furthermore, *PVT1* played a pivotal role in G1 arrest through epigenetically regulating the expression of p15 and p16 by binding to EZH2. Together, these results indicate that lncRNA *PVT1* plays a critical role in gastric cancer and may serve as a candidate target for new therapies in human gastric cancer.

## Results

### *PVT1* expression is increased in human gastric cancer tissues and correlates with poor prognosis

**Figure 1:**
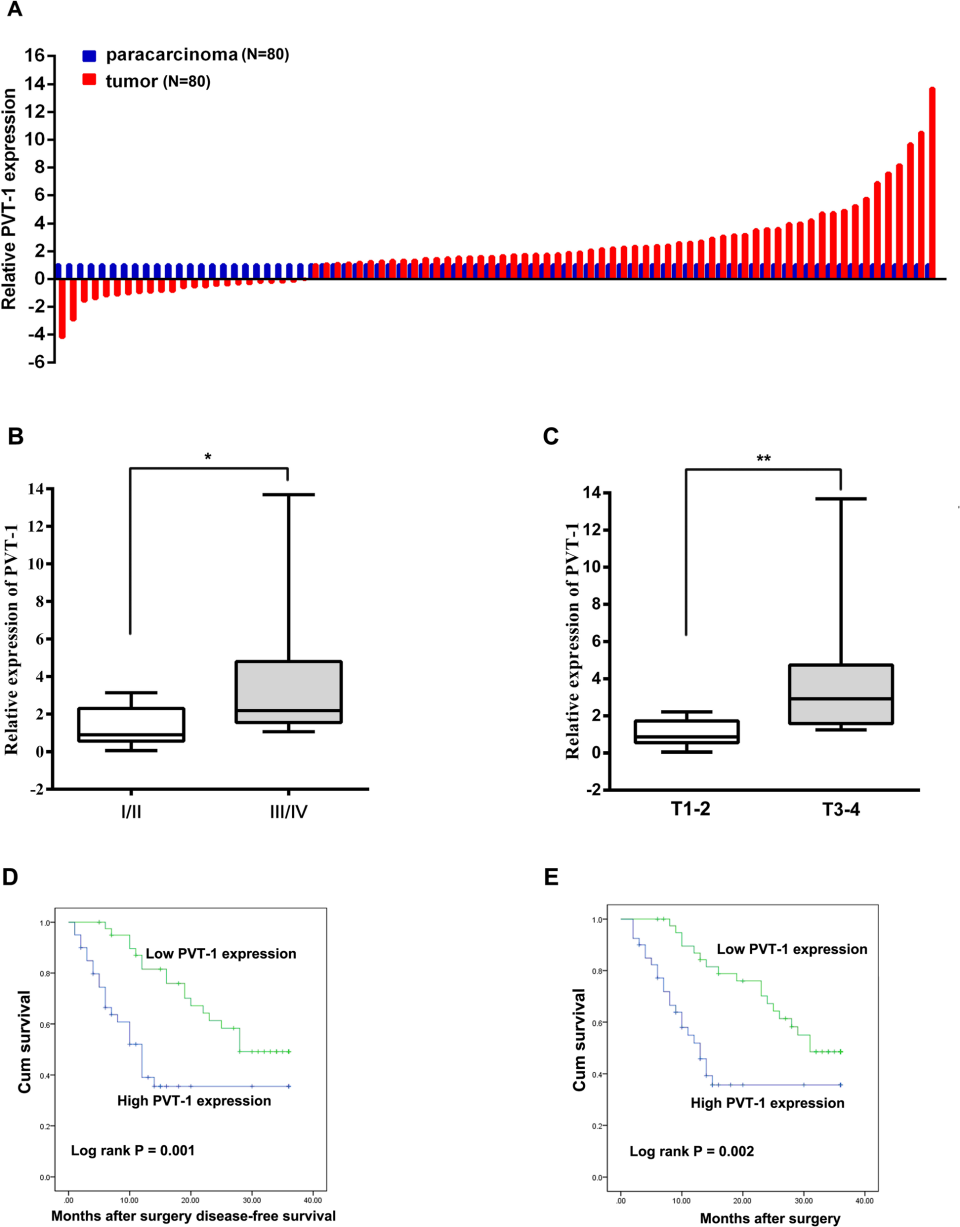
*PVT1* expression is increased in human gastric cancer tissues and correlates with poor prognosis. (**A**) Relative expression of *PVT1* in GC tissues (N = 80) compared with corresponding non-tumor tissues (N = 80). *PVT1* expression was detected by qPCR and normalized to GAPDH expression. (**B**) The *PVT1* expression was significantly higher in patients with higher pathological stage (T3-4) than in those with lower pathological stage (T1-2). (**C**) The *PVT1* expression was significantly higher in patients with deeper depth of invasion than in patients with shallower depth of invasion. Kaplan–Meier analysis of disease-free survival (**D**) or overall survival (**E**) was analyzed according to *PVT1* expression levels. *, P < 0.05, **, P < 0.01.

To investigate the role of *PVT1* in gastric cancer progression, we detected the *PVT1* expression levels in 80 paired gastric cancer tissues and corresponding non-tumor tissues by using qRT-PCR, and normalizing to GAPDH. The transcript levels of *PVT1* were significantly up-regulated in 71.25% (57 of 80) cancerous tissues compared with adjacent non-tumor tissues (p < 0.01) (Figure 1A). Next, we examined the correlation of *PVT1* expression level with patients’ clinical features in gastric cancer. As shown in Figure 1B and C, high levels of *PVT1* were correlated with advanced TNM stage (p < 0.05) and deeper invasion depth (P < 0.01). However, several other clinical parameters were found not to be significantly correlated with *PVT1* expression in our study (Table 1). The detailed results of clinical parameters and expressions were shown in Additional file 1: Table S2.

**Table 1:** **Correlation between PVT-1 expression and clinicopathological characteristics of gastric cancer**

**Clinical parameter**	***PVT1***		**Chi-squared test P-value**
	**High no. cases**	**Low no. cases**	
**Age (years)**			**0.370**
**<50**	**23**	**19**	
**>50**	**17**	**21**	
**Gender**			**0.361**
**Male**	**26**	**22**	
**Female**	**14**	**18**	
**Location**			**0.841**
**Distal**	**17**	**15**	
**Middle**	**15**	**15**	
**Proximal**	**8**	**10**	
**Histologic differentiation**			**0.206**
**Well**	**3**	**2**	
**Moderately**	**9**	**18**	
**Poorly**	**23**	**16**	
**Undifferentiated**	**5**	**4**	
**Invasion depth**			0.002*
**T1**	**5**	**14**	
**T2**	**7**	**15**	
**T3**	**14**	**6**	
**T4**	**14**	**5**	
**TNM Stages**			0.015*
**I**	**2**	**9**	
**II**	**12**	**18**	
**III**	**22**	**12**	
**IV**	**4**	**1**	
**Lymphatic metastasis**			**0.179**
**Yes**	**24**	**18**	
**No**	**16**	**22**	
**Distant metastasis**			**0.116**
**Yes**	**4**	**0**	
**No**	**36**	**40**	

*P<0.05.

To explore the relationship between *PVT1* expression and GC patients’ prognosis, we attempted to assess the correlation between *PVT1* expression and clinical outcomes. The median expression for *PVT1* in tumor tissues was used to divide the samples into high (above the median, n = 40) and low (below the median, n = 40) *PVT1* expression group. Kaplan-Meier analysis and log-rank test were performed to further evaluate the effects of *PVT1* expression and the clinicopathological characteristics on disease-free survival (DFS) and overall survival (OS). The results showed that 3 years of disease-free survival (DFS) for high *PVT1* expression is 35.5%, while is 49.2% for low *PVT1* expression. The median survival time for high *PVT1* expression is 12 months, while is 28 months for low *PVT1* expression (Figure 1D, Log rank p = 0.001). Moreover, 3 years of overall survival for high *PVT1* expression is 35.7%, while is 48.6% for low *PVT1* expression. The median survival time for high *PVT1* expression is 13 months, while is 25 months for low *PVT1* expression (Figure 1E, Log rank p = 0.002).

To further assess whether *PVT1* expression can be identified as a prognostic predictor for GC patients, the univariate and multivariate survival analyses (Cox proportional hazards regression model) were performed. By univariate analysis, we identified three prognostic factors: TNM stage (I/II, III/IV), distant metastasis and *PVT1* expression, other clinical parameters were not significant prognosis factors (Table 2). Further analysis in a multivariate Cox proportional hazards model, *PVT1* expression and TNM stage were strongly associated with DFS (p = 0.021, p = 0.023, respectively). Meanwhile, *PVT1* expression and TNM stage were also significantly correlated with OS in our study cohort (p = 0.031, p = 0.04, respectively). Taken together, these results demonstrated that *PVT1* expression was an independent prognostic indicator for DFS (HR = 2.216, 95% CI: 1.130-4.345, p = 0.021) and OS (HR = 2.092, 95% CI: 1.068-4.096, p = 0.031) in patients with gastric cancer (Table 2).

**Table 2:** **Univariate and multivariate Cox regression analyses PVT-1 for DFS or OS of patients in study cohort (n = 80)**

**Variables**	**DFS**			**OS**		
	**HR**	**95% CI**	**p value**	**HR**	**95% CI**	**p value**
**Univariate analysis**						
**Age (<50 years vs. >50 years)**	**0.830**	**0.448-1.536**	**0.552**	**0.854**	**0.462-1.581**	**0.616**
**Gender (male vs. female)**	**0.716**	**0.378-1.354**	**0.304**	**0.686**	**0.362-1.299**	**0.247**
**Location (Distal vs. Middle + Proximal)**	**0.711**	**0.383-1.322**	**0.281**	**0.668**	**0.359-1.241**	**0.201**
**Histologic differentiation (Well + Moderately vs. Poorly + Undifferentiated)**	**1.204**	**0.642-2.257**	**0.562**	**1.198**	**0.639-2.246**	**0.572**
**Invasion depth (T1 + T2 vs. T3 + T4)**	**0.629**	**0.370-1.069**	**0.087**	**0.863**	**0.466-1.597**	**0.639**
**TNM stage (I + II vs. III + IV)**	**0.377**	**0.198-0.720**	0.003*	**0.396**	**0.209-0.750**	0.004*
**Lymphatic metastasis (Yes vs. No.)**	**0.917**	**0.674-1.247**	**0.581**	**0.920**	**0.677-1.251**	**0.596**
**Regional lymph nodes (PN2+ PN3 vs. PN0+ PN1)**	**1.717**	**0.901-3.271**	**0.192**	**1.668**	**0.878-3.169**	**0.118**
**Distant metastasis (No vs. Yes)**	**0.255**	**0.073-0.892**	0.032*	**0.191**	**0.054-0.679**	0.011*
**Expression of** ***PVT1*** **(High vs. Low)**	**2.691**	**1.418-5.109**	0.002*	**2.612**	**1.381-4.942**	0.003*
**Multivariate analysis**						
**TNM stage (I + II vs. III + IV)**	**0.457**	**0.233-0.897**	0.023*	**0.496**	**0.254-0.970**	0.040*
**Lymphatic metastasis (No vs. Yes)**	**NA**			**NA**		
**Regional lymph nodes (PN0+ PN1 vs. PN2+ PN3)**	**NA**			**NA**		
**Distant metastasis (No vs. Yes)**	**0.525**	**0.145-1.899**	**0.326**	**0.371**	**0.101-1.361**	**0.135**
**Expression of** ***PVT1*** **(High vs. Low)**	**2.216**	**1.130-4.345**	0.021*	**2.092**	**1.068-4.096**	0.031*

*P<0.05.

Amplification of a region on chromosome 8q24 is one of the most frequent events in carcinomas. The well-established oncogene MYC maps to this locus and likely contributes to the pathophysiology of cancers in which it is amplified. However, the PVT1 transcript also maps to this region and has been implicated in cancer pathophysiology as well. Several published reports have revealed that aberrant PVT-1 expression caused by copynumber amplification of chromosome 8q24 [28,29]. To explore the relationship between PVT1 and 8q24 in GC, we checked the genomic amplification of PVT1-MYC region in 30 pairs GC tumor samples by qPCR. As shown in Additional file 2: Figure S2, Copy-numbers of 8q24 and PVT1 expression were positively correlated in GC tissues, and Copy-numbers of 8q24 and MYC expression were also positively correlated in GC tissues. These findings showed that 8q24 copy-number gain promoted PVT-1 expression in GC.

### Knockdown *PVT1* inhibits gastric cancer cell proliferation *in vitro*

**Figure 2:**
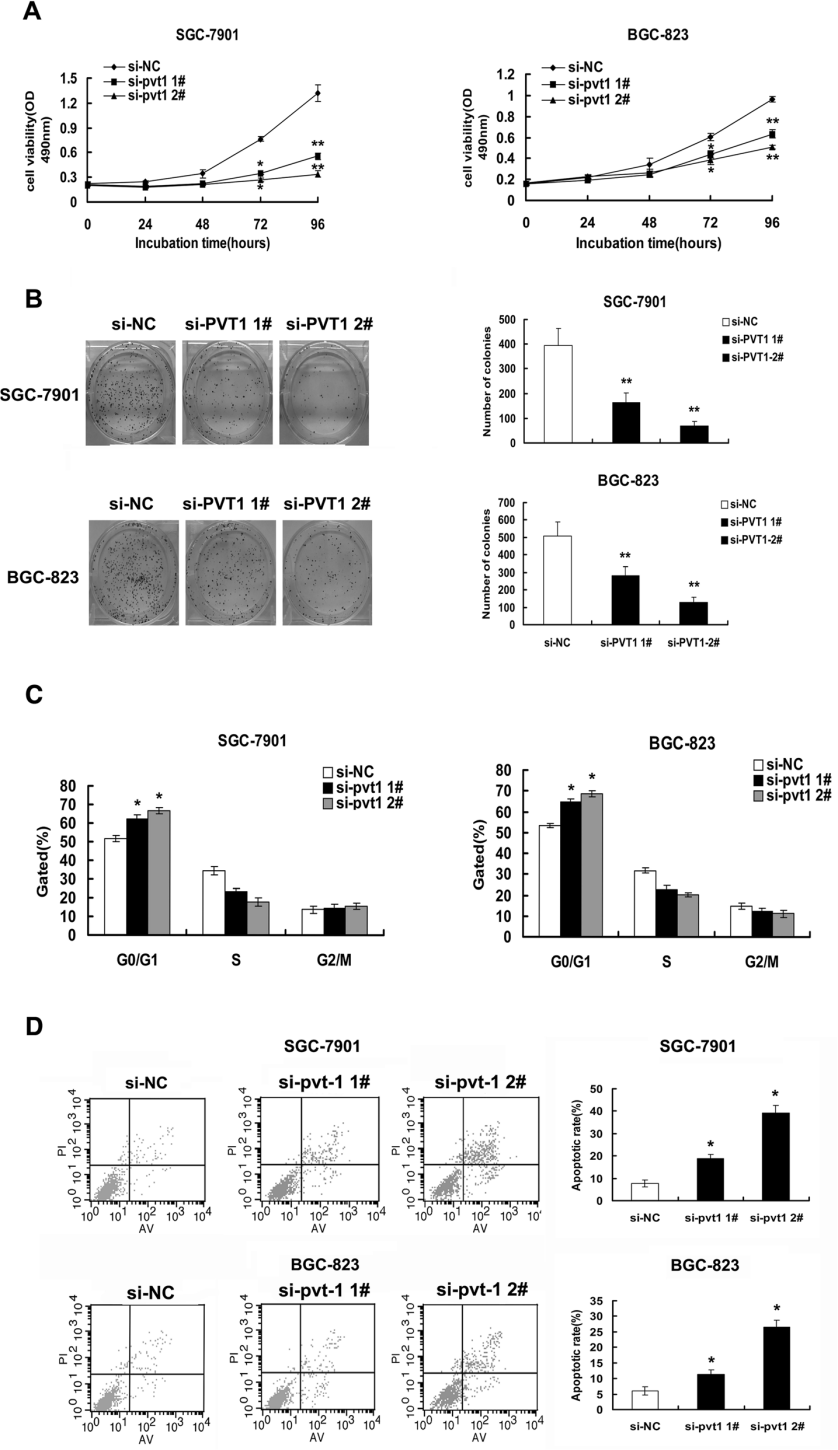
Effect of *PVT1* on gastric cell growth in vitro. (**A**) Forty-eight hours after transfection, MTT assay was performed to detect the proliferation of SGC-7901 and BGC-823 cells. (**B**) Colony-forming growth assays were performed to determine the proliferation of SGC-7901 and BGC-823 cells. The colonies were counted and captured. (**C**) Forty-eight hours after transfection, cell cycle was analyzed by flow cytometry. The bar chart represented the percentage of cells in G0/G1, S, or G2/M phase, as indicated. (**D**) Forty-eight hours after transfection, the apoptotic rates of cells were detected by flow cytometry. LR, early apoptotic cells. UR, terminal apoptotic cells. Error bars indicate means ± S.E.M. *, P < 0.05, **, P < 0.01.

To gain insight into functional role of *PVT1* in gastric cancer, qRT-PCR was performed to detect the expression of *PVT1* in GC cell lines. As shown in Additional file 3: Figure S1A, three cell lines (AGS, SGC-7901, and BGC-823) expressed higher levels of *PVT1* compared with the normal gastric epithelium cell line (GES-1). The relative high expression cell lines (SGC-7901, BGC-823) were chosen for further study. To avoid off-target effects and ensure the efficiency of interference, we used an indeed effective interference target sequence of *PVT1* according to previous study [27]. Then *PVT1* siRNA was transfected into SGC-7901 and BGC-823 cell lines. qPCR assays revealed that *PVT1* expression was significantly reduced (Additional file 3: Figure S1B). Next, MTT assay showed that knockdown of *PVT1* expression significantly inhibited cell proliferation both in SGC-7901 and BGC-823 cell lines compared with the control cells (Figure 2A). Moreover, colony-formation assay revealed that clonogenic survival was obviously decreased following inhibition of *PVT1* both in SGC-7901 and BGC-823 cell lines (Figure 2B). Furthermore, flow cytometric analysis was performed to further assess whether the effect of *PVT1* on proliferation of GC cells by affecting cell-cycle progression or apoptosis. The results showed that down-regulation of *PVT1* expression resulted in a significant increase in the percentages of cells in G1 phase compared with cells transfected with si-NC (Figure 2C). In addition, knockdown *PVT1* could obviously promote cell apoptosis (Figure 2D). These data suggested that aberrant expression of *PVT1* promoted proliferation capability of gastric cancer cells.

### The impact of *PVT1* on tumorigenesis *in vivo*

**Figure 3:**
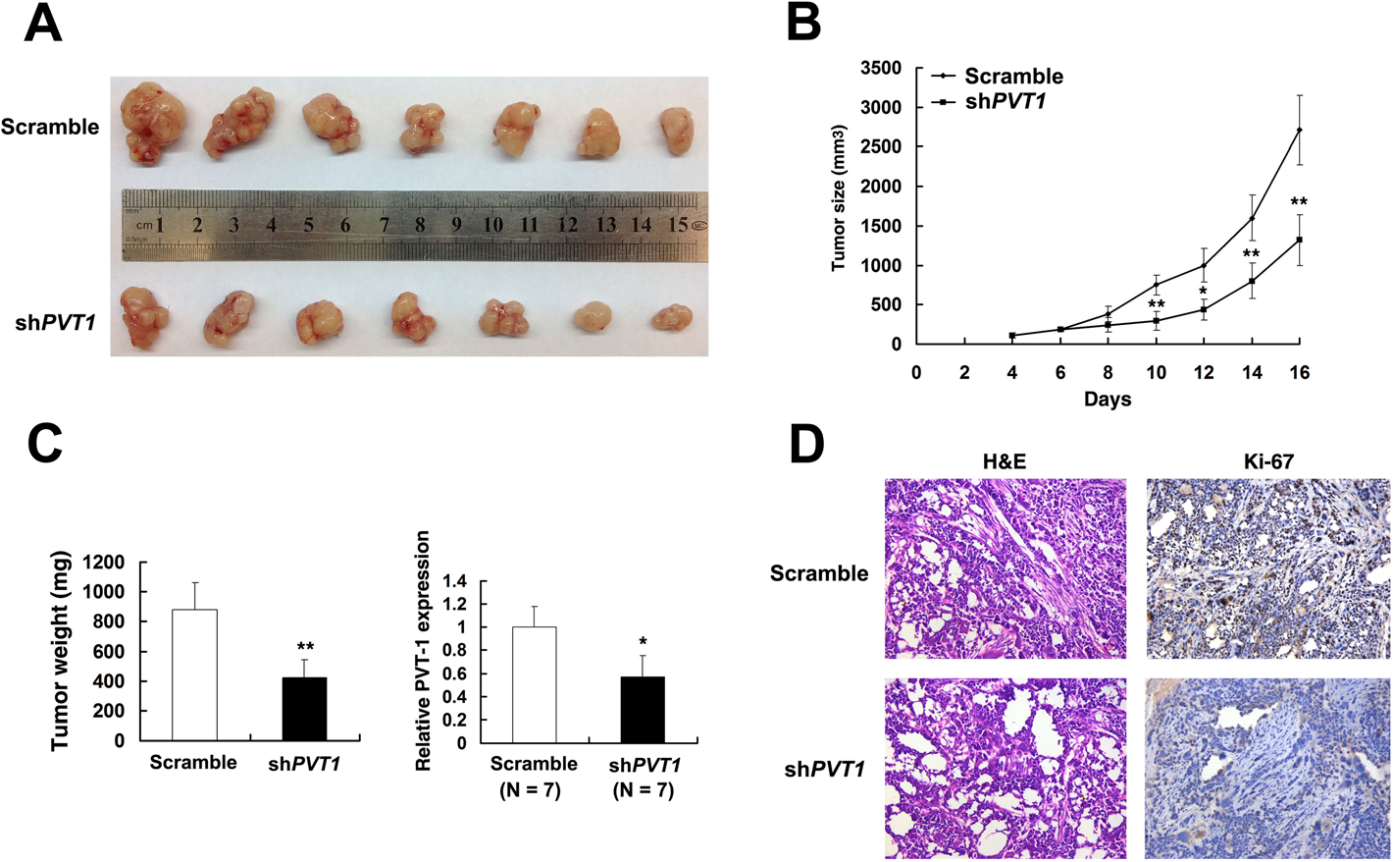
The impact of *PVT1* on tumorigenesis *in vivo*. (**A**) and (**B**) Scramble or sh*PVT1* was transfected into SGC-7901 cells, which were injected in the nude mice (n = 7), respectively. Tumor volumes were calculated every other day after 4 days of injection. Bars indicate SD. (**C**) Tumor weights are represented as means of tumor weights ± SD. qRT-PCR was performed to determine the average expression of *PVT1*. (**D**) Histopathology of xenograft tumors. The tumor sections were under H&E staining and IHC staining using antibodies against Ki-67. Error bars indicate means ± S.E.M. *, P < 0.05, **, P < 0.01.

To further determine whether the *PVT1* affects tumorigenesis, we injected SGC-7901 cells transfected with either Scramble or sh*PVT1* into nude mice. In consistent with *in vitro* results, tumor growth in sh*PVT1* group was obviously slower than that in the Scramble group (Figure 3A and B). Up to 16 days after injection, the average tumor weight in sh*PVT1* group was significantly lower than that in the control group (Figure 3C). qRT-PCR analysis was performed to detect the average expression of *PVT1* in tumor tissues. Results demonstrated that the average level of *PVT1* in sh*PVT1* group was lower than that in control group (Figure 3C). Moreover, we also found that the tumors developed from control cells showed a stronger Ki-67 expression than that in tumors formed from sh*PVT1*, as detected by IHC analysis (Figure 3D). These data further supported the role of *PVT1* in gastric cancer cell proliferation.

### *PVT1* is associated with EZH2

**Figure 4:**
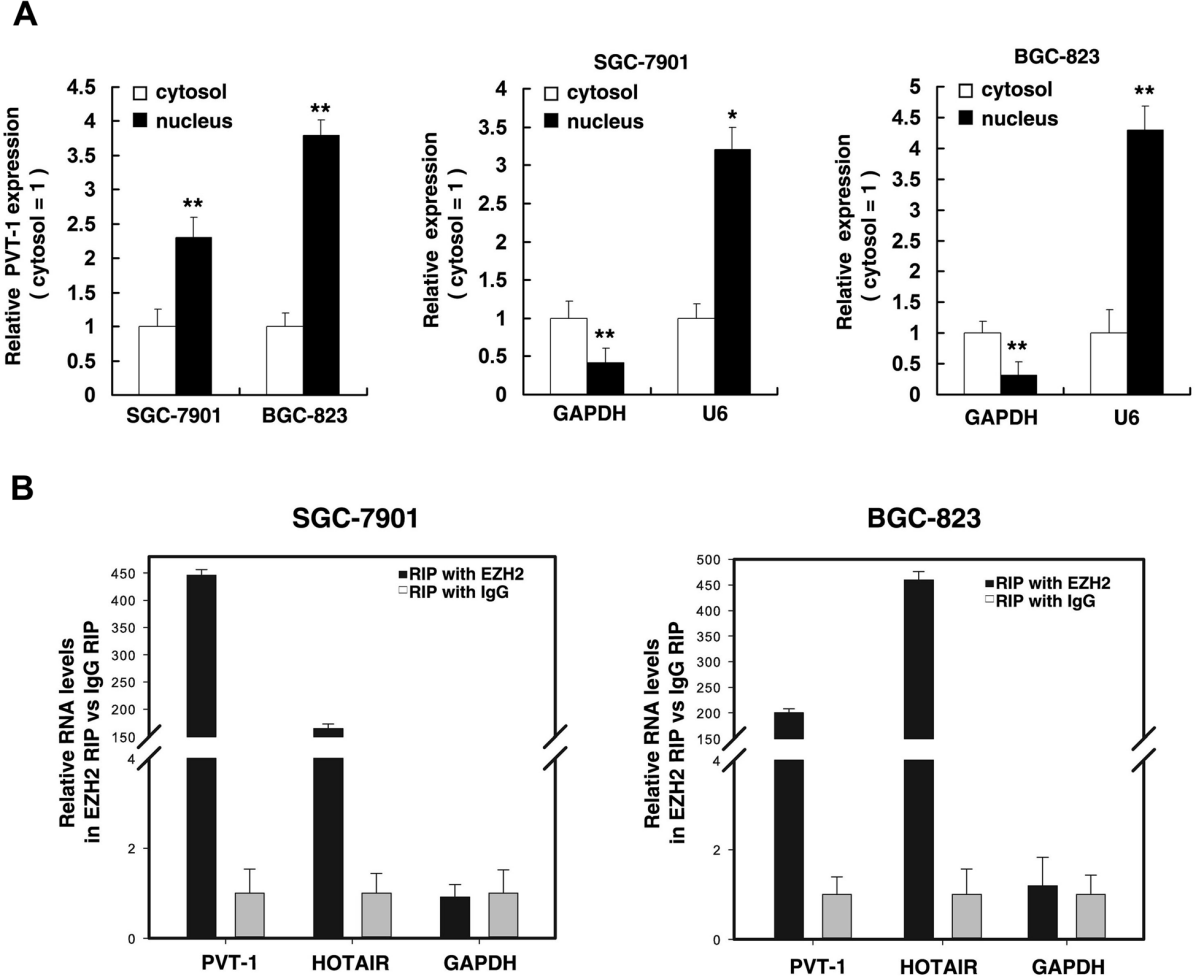
Subcellular fractionation location of *PVT1*, and *PVT1* could bind to EZH2. (**A**) After nuclear and cytosolic separation, RNA was extracted from the nuclear and the cytoplasmic fraction of SGC-7901 and BGC-823 cells and *PVT1* expression was measured by qRT-PCR. GAPDH was used as a cytosol marker and U6 was used as a nucleus marker. (**B**) RIP experiments were performed in SGC-7901 and BGC-823 cells and the coprecipitated RNA were subjected to qRT-PCR for *PVT1. HOTAIR* was used as a positive control. The fold enrichment of *PVT1* in EZH2 RIP is relative to its matching IgG control RIP. *, P < 0.05, **, P < 0.01.

Recent studies have reported that lncRNAs recruit polycomb-group proteins to specific loci and repress gene expression [12]. Twenty percent of all human lncRNAs have been shown to physically associate with Polycomb Repressive Complex 2 (PRC2 complex) [30]. Thus we hypothesized that *PVT1* might affect gene expression in such a manner. Firstly, we detected *PVT1* expression in nuclear and cytosolic fractions from SGC-7901 and BGC-823 cells by qRT-PCR. GAPDH was used as a cytosol marker and U6 was used as a nucleus marker. We found a considerable enrichment of *PVT1* in the nucleus versus the cytosol (Figure 4A), suggesting that *PVT1* expression was predominantly nuclear and *PVT1* played a major regulatory function at the transcriptional level. In addition, we performed RNA immunoprecipitation by using an antibody against enhancer of zeste homolog 2 (EZH2; an important subunit of the PRC2 complex) in SGC-7901 and BGC-823 cells. As showed in Figure 4B, the endogenous *PVT1* was enriched in the anti-EZH2 RNA immunoprecipitation (RIP) fraction relative to the input compared with the IgG fraction in SGC-7901 and BGC-823 cell lines. The endogenous lncRNA *HOTAIR*, which binds to PRC2, was used as positive control. Together, these results demonstrated a specific association between EZH2 and *PVT1*.

### *PVT1* is required to target EZH2 occupancy and activity to epigenetically regulate the expression of *p15* and *p16*

**Figure 5:**
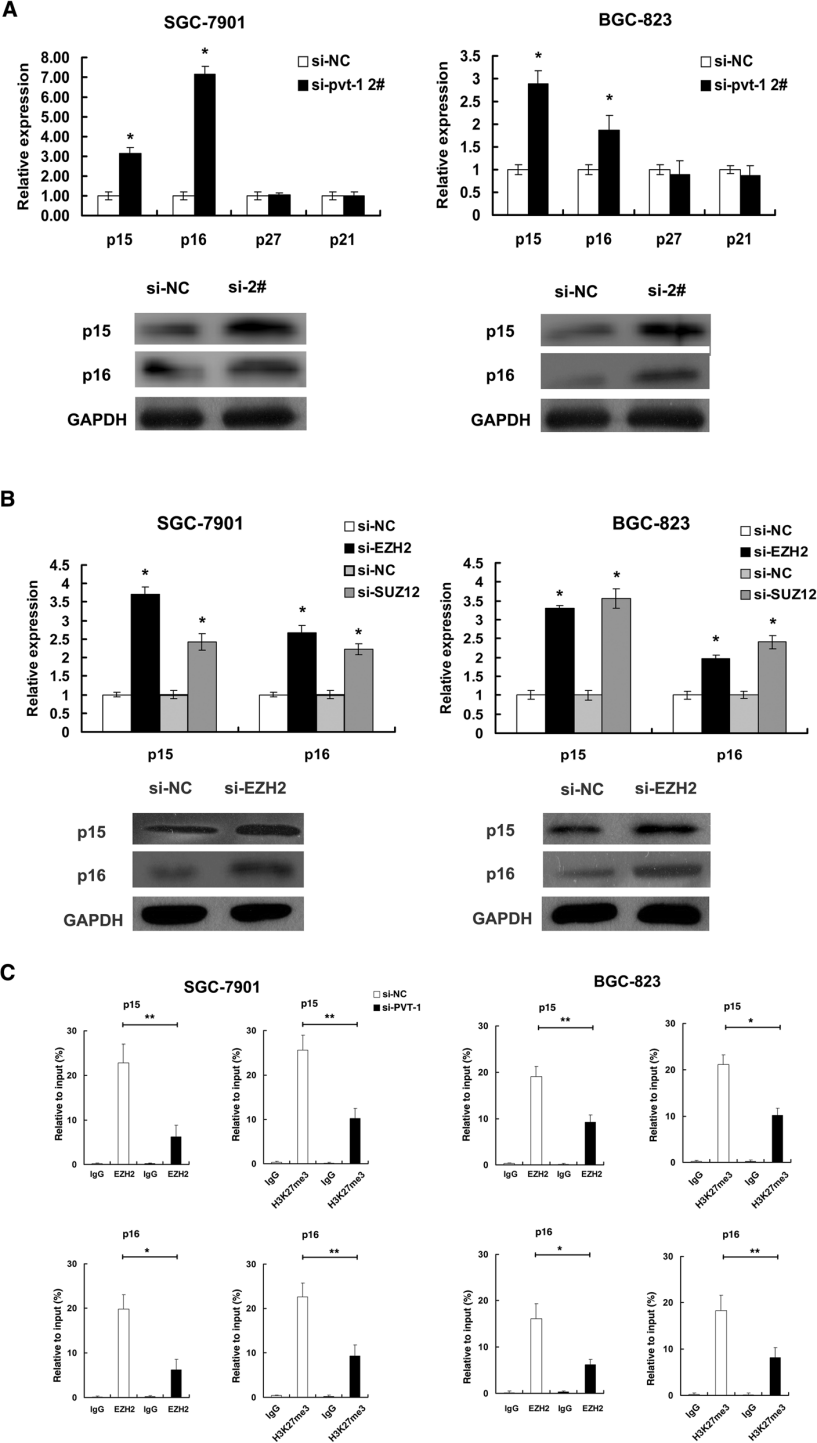
*PVT1* could regulate the expression of *p15/p16* in epigenetic level. (**A**) qPCR and western blot assays were performed to determine the expression of p15, p16, p21 and p27 in SGC-7901 and BGC-823 cells after si-*PVT1* 2# transfection. (**B**) The expression level of *p15* and *p16* was detected in SGC-7901 and BGC-823 cells after si-EZH2 or si-SUZ12 transfection by qPCR and western blot asssy was performed to detect the protein level of *p15* and *p16* after si-EZH2 transfection. (**C**) ChIP of EZH2 and H3K27me3 of the promoter region of *p15/p16* locus after siRNA treatment targeting si-NC or si-*PVT1* 2# in SGC-7901 and BGC-823 cells, qPCR was performed to detect the quantitation of ChIP assays. Enrichment was quantified relative to input controls. Antibody directed against IgG was used as a negative control. Error bars indicate means ± S.E.M. *, P < 0.05, **, P < 0.01.

To investigate the fact that *PVT1* played a role in G1/S arrest, we assessed the effect of *PVT1* inhibition on expression of cyclin-dependent protein kinase inhibitors by qRT-PCR and western blotting, and the results showed that the mRNA and protein levels of *p15, p16* were increased with the knockdown of *PVT1* (Figure 5A). The results suggested that *PVT1* may contribute to cell-cycle arrest through negatively regulating expression of the tumor suppressor *p15* and *p16*.

We then sought to determine the functional relevance of the association between *PVT1* and EZH2. We asked whether EZH2 was involved in the repression of *p15* and *p16*. The effectiveness of EZH2 and SUZ12 siRNA were presented in Additional file 3: Figure S1C. Analysis with qRT-PCR and western blotting confirmed that the expression of PRC2 target genes *p15* and *p16* [16] were increased when EZH2 was knockdown in SGC-7901 and BGC-823 cell lines (Figure 5B). Furthermore, *p15* and *p16* mRNA levels were also increased when SUZ12 (another important subunit of the PRC2 complex) was knockdown (Figure 5B).

To address whether *PVT1* is involved in transcriptional repression through enrichment of EZH2 to target gene promoters, we conducted ChIP analysis in SGC-7901 and BGC-823 cell lines. ChIP assays demonstrated that knockdown of *PVT1* decreased the binding of EZH2 and H3K27me3 levels across the *p15* and *p16* promoters compared to cells transfected with si-NC (Figure 5C). Importantly, no significant change was detected at the promoter of *HOXA9*, a target of polycomb [31] (Additional file 3: Figure S1D). These results suggest that *PVT1* is required to target EZH2 occupancy and activity to epigenetically modulate the expression of *p15* and *p16*.

### *PVT1* is inversely correlated with *p15/p16* in GC tissues

**Figure 6:**
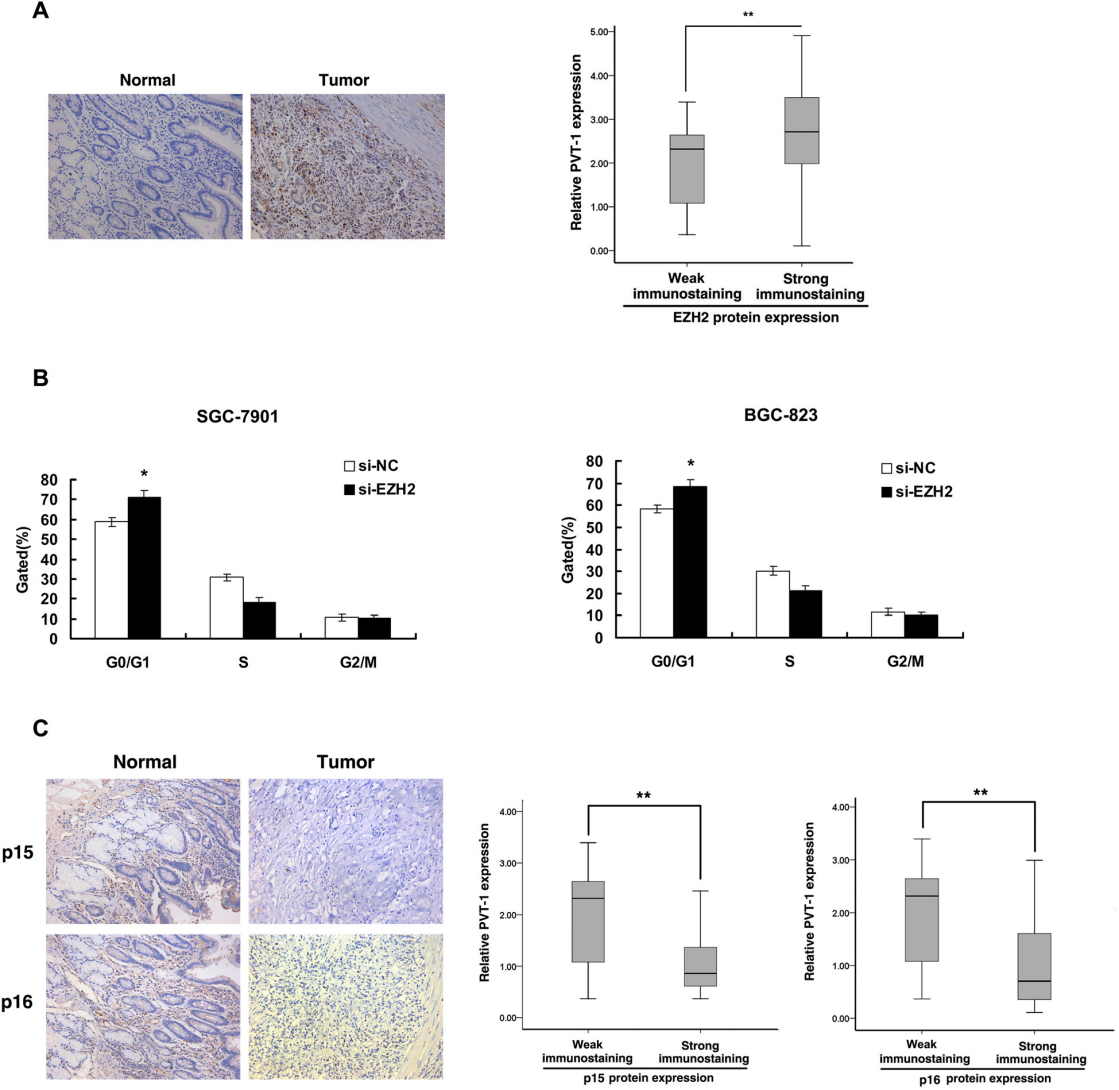
The expression of *PVT1* is inversely correlated with p15/p16 protein level in GC tissues. (**A**) Immunostaining of EZH2 was negatively or very weakly positive in non-tumor gastric tissues, but was strongly positive in corresponding tumor tissues. The immunoreactivity of EZH2 protein in GC tissues showed a statistically significant positive correlation with *PVT1* expression. Error bars indicate means ± standard errors of the mean. (**B**) Flow cytometry assays were performed to detect the cell cycle after si-EZH2 transfection. (**C**) The level of *p15/p16* in GC tissues was determined by immunohistochemistry. *PVT1* expression was inversely correlated with *p15/p16* protein level. *, P < 0.05, **, P < 0.01.

To verify the function role of EZH2 in GC, we detected the expression of EZH2 protein in 30 pairs GC tissues by IHC. Eighty percent of the tumors showed positive immunostaining of EZH2 protein. In contrast, all of the corresponding non-tumor gastric tissues showed negative or weakly positive immunostaining of EZH2 protein. Further analysis showed that the expression of *PVT1* was positively correlated with EZH2 protein level in GC tissues (Figure 6A). Moreover, flow cytometric analysis demonstrated that the cell cycle progression of si-EZH2 cells was stalled at the G1 phase compared with cells transfected with si-NC (Figure 6B). These results indicated that EZH2 was up-regulated in GC tissues and played an important role in gastric cancer cell proliferation. In addition, IHC was used to detect the expression of *p15* and *p16* proteins in gastric cancer and corresponding non-tumor gastric tissues. Most of the non-tumor gastric tissues showed strongly positive immunostaining of *p15* (20/30) and *p16* (22/30) proteins. In contrast, the corresponding GC tissues showed negative or weakly positive immunostaining of *p15* (7/30) and *p16* (5/30) proteins. Further analysis revealed that the expression of *PVT1* was inversely correlated with *p15* and *p16* protein levels in GC tissues (Figure 6C).

## Discussion

The newly discovered lncRNAs have emerged as an important player in cellular development and human diseases [32,33]. LncRNAs demonstrate temporal and tissue-specific expression patterns, and aberrant regulation in various diseases, including cancer [34].

In our present study, we demonstrated that *PVT1* expression was markedly increased in gastric cancer tissues compared with corresponding non-tumor tissues. The high expression level of *PVT1* in GC patients was associated with deeper invasion depth and advanced TNM stage. In addition, high *PVT1* expression in GC tissues was associated with a poor prognosis and could be an independent prognostic indicator. Moreover, aberrant expression of many other lncRNAs has been reported to be regarded as prognostic indicators. For example, *HOTAIR* expression is increased in primary breast tumors and metastases and its expression level in primary tumors is a powerful predictor of eventual metastasis and death [12]. Our previous studies also showed that lncRNA ANRIL and HOTAIR could serve as a prognostic factor in GC [9,35]. In addition, amplification of chromosome 8q24 is one of the most frequent events in carcinomas. The well-established oncogene MYC maps to this locus and likely contributes to the pathophysiology of cancers in which it is amplified. However, the PVT1 transcript also maps to this region and has been implicated in cancer pathophysiology as well. Several published reports have revealed that aberrant PVT-1 expression caused by copynumber amplification of chromosome 8q24. Our results showed that 8q24 copy-number gain promoted PVT-1 expression in GC and suggested that *PVT1* may play an important role in GC development, and may be useful as a novel prognostic marker for GC.

*PVT1* has been studied in a variety of physiological and pathological processes, such as diabetic nephropathy [26], colorectal cancer [27], ovarian and breast cancers [28]. However, the possible role and molecular mechanism of *PVT1* in human gastric cancer remains to be clarified. As shown in Figures 2 and 3, our results showed that *PVT1* knockdown could significantly inhibit gastric cancer cell proliferation both in vitro and in vivo. In addition, flow cytometric analysis indicated that inhibitory effect of *PVT1*-siRNA on proliferation of GC cells by causing obvious G1 phase arrest and inducing apoptosis, as shown in Figure 2.

Previous studies have reported that many lncRNAs recruit PRC2 complexes to target genes, and PRC2-mediated epigenetic regulation has an important role in cancer [12]. Twenty percent of all human lncRNAs have been shown to physically associate with Polycomb Repressive Complex 2 (PRC2 complex), suggesting that lncRNAs may have a universal role in recruiting polycomb-group proteins to their target genes [30]. In our study, cell fractionation revealed that *PVT1* was mainly localized in the nucleus, suggesting the transcriptional regulation mechanism involvement (Figure 4A). Furthermore, we performed RIP with an antibody against enhancer of zeste homolog 2 (EZH2; an important subunit of the PRC2 complex). Encouragingly, the endogenous *PVT1* was enriched in anti-EZH2 RNA immunoprecipitation (RIP) fraction relative to the input compared with the nonspecific IgG fraction both in SGC-7901 and BGC-823 cell lines (Figure 4B). Our results suggest that *PVT1* serve as a new member of PRC2-mediated epigenetic regulation and may participate in the occurrence and development of GC.

To our knowledge, the kinase activity of Cdk/cyclin complexes is tightly modulated by a plethora of Cdk inhibitors (CKIs), which serve as brakes to halt cell cycle progression [36]. Thus, we assessed the effect of *PVT1* inhibition on the expression of several CKI family proteins involved in the G1/S checkpoint in SGC-7901 and BGC-823. After inhibition of *PVT1*, we observed a significantly increase in the expression of the tumor suppressor *p15* and *p16* (Figure 5) in transcriptional level. *p15* and *p16* belong to INKs family of cyclin-dependent kinase (cdk) inhibitor proteins; they bind to cycilnD either alone or when complexed to their caralytic subunit CDK4 and prevent the activation of cyclinD-CDK4 complexes, and thus control the cell cycle progression at G1 phase. Indeed, the INK4A-ARF-INK4B gene cluster is homozygously deleted or silenced in a variety of human cancers with an estimated frequency of ~ 40%, representing one of the most frequently cytogenetic events in human cancers [37-40]. Furthermore, altered expression of *p15* and *p16* proteins was associated with GC growth [41]. These results indicated that *PVT1* could regulate proliferation of gastric cancer cells by affecting the expression of *p15* and *p16*.

Our study identified EZH2 as an important player in this *PVT1*-mediated p15 and p16 repression network because the ability of *PVT1* to repress *p15* and *p16* was dependent on the association between *PVT1* and EZH2. As shown in Figure 5B, analysis with qRT-PCR and western blotting confirmed that the expression of *p15* and *p16* were increased when EZH2 was knockdown. Previous studies have showed that *ANRIL* could epigenetically regulate *p15* and *p16* in Cis by binding to PRC2 [16]. In our study, we showed high abundance binding between *PVT1* and EZH2 in gastric cancer cells, and we further confirmed that *PVT1* could mediate epigenetic regulation of *p15* and *p16* in Trans. As shown in Figure 5C, ChIP-qPCR assays determined that *PVT1* was required for the EZH2 recruitment to and silencing of *p15* and *p16*. Moreover, overexpression of EZH2 has been described in cancers including bladder [20], gastric [21], lung [22], and hepatocellular carcinoma [24]. To further validate the role of EZH2 in gastric cancer, we detected the EZH2 expression in 30 pairs of gastric cancer tissues and corresponding adjacent tissues by IHC analysis and found that EZH2 was up-regulated in gastric cancer tissues, and the expression of EZH2 was positively correlated with *PVT1* expression (Figure 6A). Importantly, we detected a significant G1 phase arrest after si-EZH2 transfection (Figure 6B). This was consistent with the effect of *PVT1*. To determine the clinical relevance of *PVT1* and *p15/p16*, we detected *p15/p16* expression in GC tissues by IHC and found inverse relationship between the expression of *p15/p16* and *PVT1* (Figure 6C). Our finding adds a new piece (*PVT1*) to *p15* and *p16* tumor suppressor regulatory network. And p15 and p16 act as tumor suppressors in various cancers, and aberrant methylation in p15 and p16 gene promoter region has been linked to gene downregulation of expression. And PRC2-mediated histone methylation contributes to the repression of p15 and p16. Our results explained that how p15 and p16 are specifically regulated by PRC2, due to in part through *PVT1*. Taken together, we identified that *PVT1* played an oncogenic role in gastric cancer partly through epigenetic regulation of *p15* and *p16*.

In a broader perspective, the identification of *PVT1* as an important prognostic factor for GC patients calls our attention to exploring its functional roles. Moreover, *PVT1* could regulate gastric cancer cells proliferation both *in vitro* and *in vivo*. Importantly, we first reported that *PVT1* serving as a member of PRC2-mediated epigenetic regulation participated in the development of gastric cancer. Our study may provide a strategy and facilitate the development of lncRNA directed diagnostics and therapeutics against this deadly disease.

## Materials and methods

### Cell culture

Three gastric cancer cell lines (AGS, SGC-7901, BGC-823), and a normal gastric epithelium cell line (GES-1) were purchased from the Institute of Biochemistry and Cell Biology of the Chinese Academy of Sciences (Shanghai, China). Cells were cultured in RPMI 1640 or DMEM with 10% FBS (Gibco), and cultured at 37°C with 5% CO2.

### Tissue samples and clinical data collection

Human gastric cancer specimens were obtained from the First Affiliated Hospital of Nanjing Medical University. This study was approved by Research Ethics Committee of Nanjing Medical University (Nanjing, Jiangsu, PR China). Written informed consent was obtained from all patients. Corresponding normal gastric tissue samples were taken from tissues that were located 5 cm away from tumor margin. The characteristics of the patients were summarized in Table 1.

### RNA extraction and qRT-PCR analyses

Total RNA was extracted from gastric cancer tissues or cells by using TRIzol reagent (Invitrogen), according to the manufacturer’s protocol. RNA was reverse transcribed to cDNA by using a Reverse Transcription Kit (Takara, Dalian, China). Real-time PCR was performed with SYBR Green (Takara, Dalian China). GAPDH was used as reference for mRNA or lncRNAs. Each sample was analyzed in triplicate. The primers were listed in Additional file 4: Table S1.

### RNA interference

Gastric cancer cells were transfected with siRNA by using Lipofectamine 2000 (Invitrogen, USA), according to the manufacturer’s protocol. The cells were incubated for 48 h before use in assays. The siRNA sequences were listed in Additional file 4: Table S1. The shRNA *PVT1* (CCCAACAGGAGGACAGCTT) was cloned into pENTR™/U6 vector.

### Cell proliferation assays

Cell proliferation was tested with MTT kit (Sigma) according to the manufacturer’s instruction. For colony formation assay, a certain number of transfected cells were placed in each well of a six-well plate and maintained in proper media containing 10% FBS for two weeks, during which the medium was replaced every 4 days. Colonies were then fixed with methanol and stained with 0.1% crystal violet (Sigma) in PBS for 10 minutes. Colony formation was determined by counting the number of stained colonies.

### Flow-cytometric analysis

Transfected cells were harvested after 48 h transfection. After the double staining with fluorescein isothiocyanate (FITC)-Annexin V and propidium iodide was done by the FITC Annexin V Apoptosis Detection Kit (BD Biosciences) according to the manufacturer’s recommendations. The cells were analyzed with a flow cytometry (FACScan; BD Biosciences) equipped with a Cell Quest software (BD Biosciences). Cells were discriminated into viable cells, dead cells, early apoptotic cells, and apoptotic cells, and then the relative ratio of early apoptotic cells were compared with control transfection from each experiment. Cells for cell-cycle analysis were stained with propidium oxide by the CycleTEST PLUS DNA Reagent Kit (BD Biosciences) following the protocol and analyzed by FACScan. The percentage of the cells in G0–G1, S, and G2–M phase were counted and compared.

### Western blot assay and antibodies

Cells protein lysates were separated by 15% SDS-polyacrylamide gel electrophoresis (SDS-PAGE) transferred to 0.22 μm NC membranes (Sigma) and incubated with specific antibodies. GAPDH antibody was used as control. Autoradiograms were quantified by densitometry (Quantity One software; Bio-Rad). Anti-p15 and anti-p16 were purchased from Santa Cruz Biotechnology, Inc. Anti-Ki67 was purchased from Santa Cruz Biotechnology.

### Subcellular fractionation location

The separation of nuclear and cytosolic fractions was performed using the PARIS Kit (Life Technologies, Carlsbad, CA, USA) according to the manufacturer’s instructions.

### RNA immunoprecipitation (RIP)

RNA immunoprecipitation(RIP) experiments were performed by using a Magna RIP™ RNA-Binding Protein Immunoprecipitation Kit (Millipore, USA) according to the manufacturer’s instructions. Antibody for RIP assays of EZH2 was from Abcam.

### Chromatin immunoprecipitation (ChIP) assays

ChIP assays were performed using EZ-CHIP KIT according to the manufacturer’s instruction (Millipore, USA). EZH2 antibody was obtained from Abcam. H3 trimethyl Lys 27 antibody was from Millipore. The ChIP primer sequences were listed in Additional file 4: Table S1. Quantification of immunoprecipitated DNA was performed using qPCR with SYBR Green Mix (Takara). ChIP data was calculated as a percentage relative to the input DNA by the equation 2[Input Ct − Target Ct] × 0.1 × 100.

### Animal work

Animal work was performed according to the procedures as previously described [9]. 5-week-old male athymic BALB/c mice were maintained under specific pathogen-free conditions and manipulated according to protocols approved by the Shanghai Medical Experimental Animal Care Commission. SGC-7901 cells transfected with Scramble or sh*PVT1* were harvested at a concentration of 2 × 10^7^ cells/ml. Of the suspending cells, 0.1 ml was subcutaneously injected into the flanks of the nude mouse, one injection per mouse. Tumor growth was monitored, and tumor sizes and weights were measured every two days. Tumor volume was calculated using the formula, volume = (length × width^2^ × 0.5). Sixteen days after injection, the mice were killed and tumor weights were measured and used for further analysis. The primary tumors were excised and tumor tissues were used to perform qRT-PCR analysis of *PVT1* expression levels and immunostaining analysis of Ki-67 protein expression.

### Immunohistochemistry (IHC)

The primary tumors were immunostained for Ki-67 as previously described [17].

### Statistical analysis

All statistical analyses were performed by using SPSS 20.0 software (IBM, SPSS, USA). The significance of differences between groups was estimated by Student’s *t*-test, *χ*2 test or Wilcoxon test, as appropriate. DFS and OS rates were calculated by the Kaplan-Meier method with the log-rank test applied for comparison. Survival data were evaluated using univariate and multivariate Cox proportional hazards model. Variables with a value of p < 0.05 in univariate analysis were used in subsequent multivariate analysis on the basis of Cox regression analyses. Two-sided p-values were calculated, and a probability level of 0.05 was chosen for statistical significance.

## Additional files

Additional file 1: Table S2. The detailed results of clinical parameters and expressions. Additional file 2: Figure S2. qPCR was used to check relationship between the genomic amplification of 8q24 and expression of PVT1/MYC in 30 pairs GC samples. Additional file 3: Figure S1. Supplementary experimental results. (A) *PVT1* was detected in GC cells by qRT-PCR, data was presented as fold-change in GC cell lines (AGS, SGC-7901, BGC-823) relative to GES-1 cell line. (B) The relative expression level of *PVT1* in SGC-7901 and BGC-823 cells, transfected with si-NC or si-*PVT1* (si-*PVT1* 1#, 2#), was tested by qPCR. (C) The relative expression level in SGC-7901 and BGC-823 cells, after knockdown EZH2 and SUZ12, was tested by qPCR. (D) ChIP-qPCR of H3K27me3 and EZH2 of the promoter region of HOXA9 after siRNA treatment targeting si-NC or si-*PVT1* in SGC-7901 cells. Antibody enrichment was quantified relative to the amount of input DNA. *, P < 0.05, **, P < 0.01. Additional file 4: Table S1. The list of primers and the sequence of siRNAs.
