# Supplementary figures and images for: Long noncoding RNA PVT1 indicates a poor prognosis of gastric cancer and promotes cell proliferation through epigenetically regulating p15 and p16
Source: Mol Cancer. 2015 Apr 12;14:82. doi: 10.1186/s12943-015-0355-8 (PMC4399399; doi:10.1186/s12943-015-0355-8)

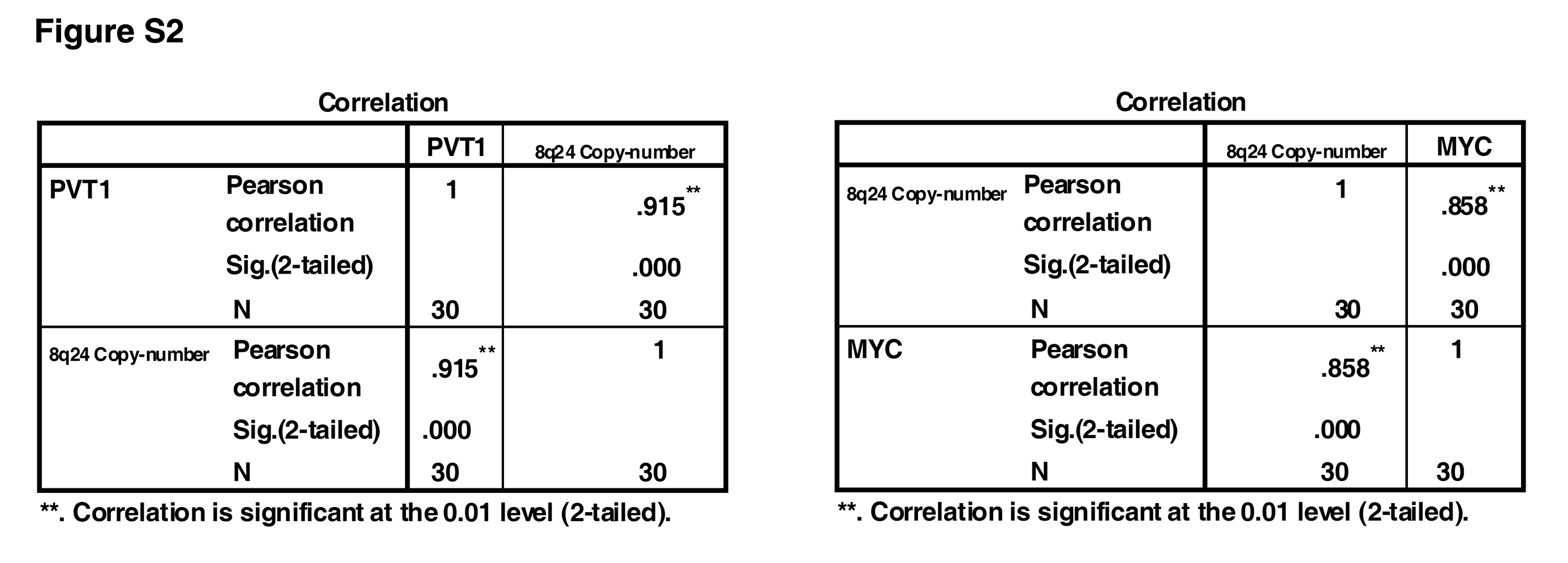

Supplement: Additional file 2: Figure S2. — qPCR was used to check relationship between the genomic amplification of 8q24 and expression of PVT1/MYC in 30 pairs GC samples. [file 12943_2015_355_MOESM2_ESM.tiff]

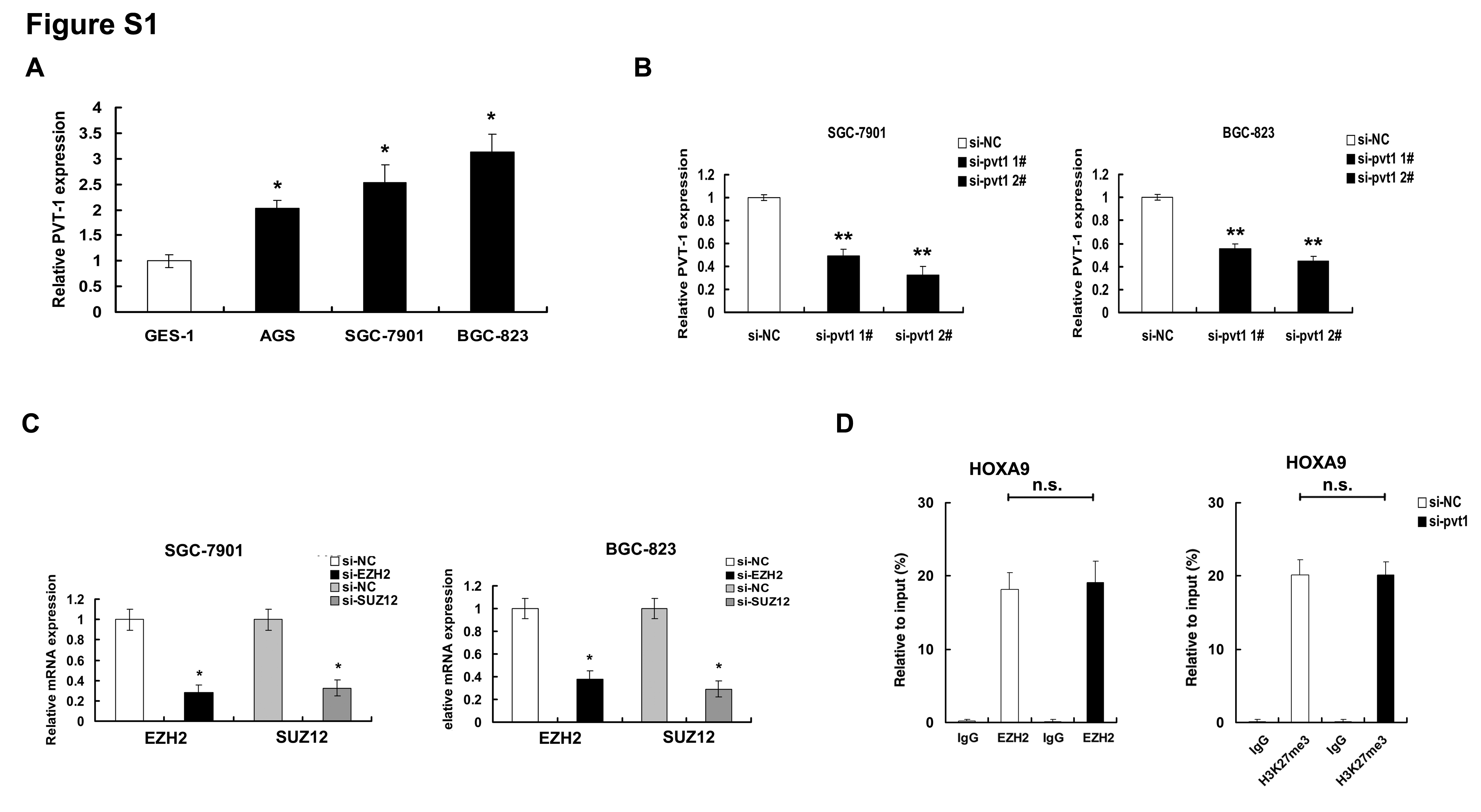

Supplement: Additional file 3: Figure S1. — Supplementary experimental results. (A) PVT1 was detected in GC cells by qRT-PCR, data was presented as fold-change in GC cell lines (AGS, SGC-7901, BGC-823) relative to GES-1 cell line. (B) The relative expression level of PVT1 in SGC-7901 and BGC-823 cells, transfected with si-NC or si-PVT1 (si-PVT1 1#, 2#), was tested by qPCR. (C) The relative expression level in SGC-7901 and BGC-823 cells, after knockdown EZH2 and SUZ12, was tested by qPCR. (D) ChIP-qPCR of H3K27me3 and EZH2 of the promoter region of HOXA9 after siRNA treatment targeting si-NC or si-PVT1 in SGC-7901 cells. Antibody enrichment was quantified relative to the amount of input DNA. *, P < 0.05, **, P < 0.01. [file 12943_2015_355_MOESM3_ESM.tiff]
