# Supplementary material for: Long noncoding RNA PVT1 indicates a poor prognosis of gastric cancer and promotes cell proliferation through epigenetically regulating p15 and p16
Source: Mol Cancer. 2015 Apr 12;14:82. doi: 10.1186/s12943-015-0355-8 (PMC4399399; doi:10.1186/s12943-015-0355-8)
Supplement: Additional file 4: Table S1. — The list of primers and the sequence of siRNAs. [file 12943_2015_355_MOESM4_ESM.doc]

**Table S1 The list of primers and the sequence of siRNAs**

| **GENE** | **Forward primer** | **Reverse primer** |
| --- | --- | --- |
| **human qRT-PCR** | |  |
| **PVT-1** | **TGAGAACTGTCCTTACGTGACC** | **AGAGCACCAAGACTGGCTCT** |
| **P15** | **GGACTAGTGGAGAAGGTGCG** | **GGGC GCTGCCCATCATCATG** |
| **P16** | **CACCGAATAGTTACGGTCGG** | **GCACGGGTCGGGTGAGAGTG** |
| **P21** | **AGACCATGTGGACCTGTCACTG** | **GTTTGGAGTGGTAGAAATCTGTC** |
| **P27** | **TGCAACCGACGATTCTTCTACTCAA** | **CAAGCAGTGATGTATCTGATAAACAAGG** |
| **EZH2** | **TGCACATCCTGACTTCTGTG** | **AAGGGCATTCACCAACTCC** |
| **SUZ12** | **TGCAGTTCACTCTTCGTTGG** | **TGCTTCAGTTTGTTGCCTTG** |
| **U6** | **CTCGCTTCGGCAGCACA** | **AACGCTTCACGAATTTGCGT** |
| **HOTAIR** | **CAGTGGGGAACTCTGACTCG** | **GTGCCTGGTGCTCTCTTACC** |
| **GAPDH** | **AGCCACATCGCTCAGACAC** | **GCCCAATACGACCAAATCC** |
| **siRNA序列** | |  |
| **si-PVT-1 1#** | **GCUUGGAGGCUGAGGAGUUTT** | **AACUCCUCAGCCUCCAAGCTT** |
| **si-PVT-1 2#** | **CCCAACAGGAGGACAGCUUTT** | **AAGCUGUCCUCCUGUUGGGTT** |
| **si-EZH2** | **GAGGUUCAGACGAGCUGAUUU** |  |
| **si-SUZ12** | **GUCGCAACGGACCAGUUAA** |  |
| **ChIP qPCR primers** | |  |
| **P15** | **TCTGGTAAGGGTGTGCTGTG** | **AAAACTCCTCTGTGGCATGTG** |
| **P16** | **AGGGGAAGGAGAGAGCAGTC** | **GGGTGTTTGGTGTCATAGGG** |
| **HOXA9** | **TGACCTTGAATGGCCCAAAG** | **CTGGTCACAGCAGGTAGGGTTAG** |
| **ShPVT1 :Sequences cloned into pENTR™/U6 vector** | |  |
| **CACCGCCCAACAGGAGGACAGCTTCGAAAAGCTGTCCTCCTGTTGGG** | | **AAAACCCAACAGGAGGACAGCTTTTCGAAGCTGTCCTCCTGTTGGGC** |
